# Supplementary material for: A Minimalistic Resource Allocation Model to Explain Ubiquitous Increase in Protein Expression with Growth Rate
Source: PLoS One. 2016 Apr 13;11(4):e0153344. doi: 10.1371/journal.pone.0153344 (PMC4830519; doi:10.1371/journal.pone.0153344)
Supplement: S1 Text — (PDF) [file pone.0153344.s001.pdf]

S1 Text

Effects of degradation and varying synthesis rate on model predictions
The predicted fraction of an unregulated protein as a function of the growth rate is to follow a linear trend crossing at the origin (S1 Fig, blue dots). Degradation can be interpreted as implying that the observed growth rate is the combination of the bio-synthesis rate minus the degradation rate, implying that the predicted fraction of an unregulated protein is linear increase with growth rate, but with a horizontal intercept at minus the degradation rate as is shown by the green dots in S1 Fig.

Non constant bio-synthesis rate can be modeled as a Michaelis-Menten kinetic like interdependence with growth rate following the formula:

$$\eta(g(c)) = \frac{\eta_0}{1 + \frac{g_m}{g(c)}} \tag{S1}$$

where $g(c)$ is the growth rate, $\eta(g(c))$ is the bio-synthesis rate at growth rate $g(c)$, η_0 is the maximal bio-synthesis rate and g_m the growth rate at which the bio-synthesis rate is $\frac{1}{2}$ the maximal rate (S1 Fig, red line). Under this assumption, the doubling time of the bio-synthesis machinery itself, T_B becomes:

$$T_B = T_B^0 \frac{\eta(g(c))}{\eta_0} = T_B^0 (1 + \frac{g_m}{g(c)}) \tag{S2}$$

where T_B^0 is the minimal theoretical doubling time when all the proteins are bio-synthesis proteins operating at maximal rate. Substituting Eq.(S2) into Eq.(4) results in a predicted fraction of:

$$p_i(c) = \frac{w_i(c)}{W_B} \frac{T_B^0 (1 + \frac{g_m}{g(c)})}{\ln(2)} g(c) = \frac{w_i(c)}{W_B} \frac{T_B^0}{\ln(2)} (g(c) + g_m) \tag{S3}$$

Surprisingly, this equation also describes the fraction as being linearly dependent on the growth rate, with the kinetic parameters implying a non-zero fraction at zero growth rate as is shown by the red dots in S1 Fig.
