# Supplementary material for: A Minimalistic Resource Allocation Model to Explain Ubiquitous Increase in Protein Expression with Growth Rate
Source: PLoS One. 2016 Apr 13;11(4):e0153344. doi: 10.1371/journal.pone.0153344 (PMC4830519; doi:10.1371/journal.pone.0153344)
Supplement: S2 Text — (PDF) [file pone.0153344.s003.pdf]

S2 Text

Datasets omitted from the main analysis Two published whole proteome *E.coli* data sets were omitted from our analysis ([24] and [25]). Both of these data sets present gradual modulation of the growth rate by a single factor (the data set from [25] uses 3 different factors, each independently). Such modulations yield very typical distributions of correlation with growth rate of proteins roughly dividing the proteome to 3 groups, proteins the fraction of which increases with growth rate, proteins the fraction of which decreases with growth rate, and proteins that show no evident correlation with growth rate (S2 Fig).

Under such conditions it is evident that one cannot distinguish between proteins that are actively up-regulated, and proteins that are passively regulated, as in either case (by our predictions) such proteins will increase in fraction with growth rate. Furthermore, smaller number of conditions increases the probability of proteins to yield extremely high or low correlation with growth rate due to measurement noise. To illustrate, in the extreme case of measuring only two conditions, every protein (unless presenting exactly the same amount under the two conditions) will have either a perfect positive correlation with the growth rate, or a perfect negative correlation with the growth rate, depending on whether it is highly expressed under the second condition or the first one. Unfortunately, both of these data sets use relatively small number of measurements.

The data sets from [25] use different titration methods to modify the growth rate under different limitations. It is unclear how the regulatory network of the cells responds to these titration methods as they use artificial constructs or antibiotics, both of which are signals the regulatory network likely did not evolve to respond to.

The range of growth rate spanned by the data set from [25] is relatively narrow. From our analysis of the other data sets, it follows that the predicted changes in fraction due to passive effects under such a range are small, making it hard to distinguish between actual changes and measurement noise.

Finally, the data set from [25] does not report absolute protein quantities but rather the ratio of protein change to a standard condition of growth. It therefore does not allow us to estimate the magnitude of changes in terms of mass and total fractions of the proteome as is needed by our analysis.
