# Supplementary material for: A Minimalistic Resource Allocation Model to Explain Ubiquitous Increase in Protein Expression with Growth Rate
Source: PLoS One. 2016 Apr 13;11(4):e0153344. doi: 10.1371/journal.pone.0153344 (PMC4830519; doi:10.1371/journal.pone.0153344)
Supplement: S3 Text — (PDF) [file pone.0153344.s005.pdf]

S3 Text

Correlation between the different proteomics data sets To assess the agreement between the different data sets we used, as well as those we omitted, we compared the correlation with growth rate of each protein under similar conditions between every two of these data sets.

In the data set from [29] we used the 4 glucose limited chemostat growth conditions. In the data set from [13] we used the 9 glucose accelerostat growth conditions. In the data set from [24] we used all 5 glucose accelerostat growth conditions. In the data set from [25] we used each limiting set separately. S3 Fig shows the relations between all of these data sets.

The glucose limited continuous growth sets show a covariance of $\approx 0.4 - 0.5$ between each other. A slightly lower covariance of $\approx 0.3 - 0.4$ is observed when comparing each of the glucose limited continuous cultures to the carbon limited data from [25] Moving on to nitrogen limiting conditions compared with continuous growth, the covariance drops to ≈ 0.2. Finally, the translation limiting conditions from [25] present a small negative covariance with all of the other conditions.

These results imply that indeed the different limitations imposed in [25] expose different regulatory mechanisms than the ones at play under standard carbon limited growth. They also highlight the fact that different data sets, despite being measured under similar growth conditions, present large differences. This analysis emphasizes the importance of analyzing each data set separately as consistency between data sets from different labs is not very high.
