# Supplementary material for: A Minimalistic Resource Allocation Model to Explain Ubiquitous Increase in Protein Expression with Growth Rate
Source: PLoS One. 2016 Apr 13;11(4):e0153344. doi: 10.1371/journal.pone.0153344 (PMC4830519; doi:10.1371/journal.pone.0153344)
Supplement: S4 Text — (PDF) [file pone.0153344.s007.pdf]

S4 Text

Threshold choice for defining strong correlation with growth rate The data we use includes the fractions of proteins under different growth conditions, and the growth rate for every condition. We select a threshold correlation with growth rate to define the group of highly positively correlated with growth rate proteins.

We calculate the explained variability by the growth rate, given a threshold, by taking the difference between the total variability of the group of proteins with a correlation higher than the threshold, and the variability remaining, when assuming these proteins scale with the growth rate according to the calculated linear response. Dividing the explained variability by the total variability of the entire data set quantifies what fraction of the total variability in the proteome is explained by considering a coordinated linear scaling with growth rate for all the proteins with a correlation with growth rate higher than the threshold.

The choice of threshold is thus influenced by two contradicting factors. Choosing a low threshold results in defining many proteins as being highly positively correlated with growth rate. In this case, the correlation with growth rate of these proteins spans a large range. Therefore, applying a linear regression trend to the sum of these proteins only accounts for a small fraction of the variability of them and, as a consequence, only accounts for a small fraction of the total variability of the proteome.

On the other hand, choosing a high correlation threshold results in defining only a small number of proteins as being highly positively correlated with growth rate. A common linear regression line may thus explain a large fraction of the variability for the chosen proteins but, as their number is small, will only account for a small fraction of the total variability of the proteome.

For simplicity, we chose a threshold value of 0.5 for the two data sets analyzed in this study. S4 Fig shows how the choice of threshold affects the fraction of explained variability in the proteome by the linear dependence on growth rate of the proteins that have a correlation with growth rate that is higher than the threshold (blue line). The figure also shows the fraction of proteins that have a correlation with growth rate that is higher than the threshold out of the proteome (red line), and the fraction of explained variability by linear regression for these proteins (green line).

The optimal threshold is defined as the threshold maximizing the fraction of total variability explained (maximum of the blue line). As can be seen in S4 Fig, our choice of threshold of 0.5 is relatively close to the optimum value that is 0.25 for the data set from [29], and 0.2 for the data set from [13]. Moreover, as S4 Fig illustrates, the different plotted statistics do not change markedly due to this sub-optimal choice of threshold and thus this choice does not affect our results significantly.

As different proteins have very different average fractions, the aforementioned calculation may be biased towards proteins with higher average fractions. To avoid this effect, the analysis presented was performed on the normalized fractions as defined in the Methods section.

The noise in current whole proteome measurement techniques makes it difficult to distinguish between proteins that scale coordinately, as is predicted by our model, and proteins that scale differentially, but within measurement uncertainty. Thus, it is unclear to what extent the effect we predict affects actual protein fractions versus their possible individual up regulation with growth rate. We expect future improvements in the accuracy of whole proteome measurements to quantitatively reveal the importance of passive coordinated scaling with growth rate in shaping the proteome composition. These coming improvements in accuracy will enable better testing of the scope and validity of the model presented here.
