# Supplementary material for: A Minimalistic Resource Allocation Model to Explain Ubiquitous Increase in Protein Expression with Growth Rate
Source: PLoS One. 2016 Apr 13;11(4):e0153344. doi: 10.1371/journal.pone.0153344 (PMC4830519; doi:10.1371/journal.pone.0153344)
Supplement: S5 Text — (PDF) [file pone.0153344.s009.pdf]

S5 Text

Calculation of expected distribution given experimental noise and deviation of maximum between between the expected distribution and the observed one The expected distribution of slopes was calculated as follows: For every protein that was identified as being strongly positively correlated with growth rate, a linear regression with growth rate was performed, and the normalized slope and standard error of the regression line were calculated. The average standard error and average slope across all proteins that were identified as being strongly positively correlated with growth rate were calculated. Denoting by n the number of conditions, we note that each slope is a statistical variable calculated based on n samples, with $n-2$ degrees of freedom. The calculated slopes of the different proteins are therefore expected to follow a student-t distribution with $n-2$ degrees of freedom, a standard deviation that is the average of the standard errors calculated for the regression of each protein, and an average value that is equal to the average taken over all of the calculated slopes. The average slope, average standard error and $n-2$ degrees of freedom were therefore used to calculate the expected student-t distribution of slopes that would emerge, should all proteins share the same average slope with the given average standard error in measurements.

While the expected distributions of slopes are symmetric, the observed ones are asymmetric, with a longer distribution tail at higher slopes. This is a side effect of the way by which proteins were identified to be strongly positively correlated with growth rate, given an experimental noise. To understand why, one must consider the effect of noise on proteins with shallow slopes versus proteins with steep slopes. Proteins with shallow slopes present small change in level across growth rates. Thus, when experimental noise is present, its effect on variability of protein levels is large compared with the changes in level resulting from the dependence on growth rate. Therefore, the Pearson correlation with growth rate of such proteins is more severely affected by noise, compared with proteins with a steep slope. As a result, proteins with shallow slopes face a higher chance of presenting low correlation with growth rate, resulting in them not being identified as proteins with high correlation with growth rate. Such mis-classification, in turn, causes such proteins to be under-represented in the set of proteins which are highly correlated with growth rate.

To conclude, the proteins identified as strongly positively correlated with growth rate are most of the proteins with steep slopes, but only the proteins with shallow slopes that suffered small experimental noise, resulting in an enrichment of higher slopes and an asymmetric distribution of observed slopes. With this, the average observed slope is expected to be higher than the real one.

The calculated expected distribution does not take these effects into account as it only presents the expected distribution of slopes, while not subtracting proteins that present a correlation with growth rate that is smaller then the threshold defined. It therefore presents a symmetric distribution with a mean value that appears to be higher than the one observed due to the difference in symmetries between the two distributions.
