# Supplementary material for: A Minimalistic Resource Allocation Model to Explain Ubiquitous Increase in Protein Expression with Growth Rate
Source: PLoS One. 2016 Apr 13;11(4):e0153344. doi: 10.1371/journal.pone.0153344 (PMC4830519; doi:10.1371/journal.pone.0153344)
Supplement: S6 Text — (PDF) [file pone.0153344.s010.pdf]

## S6 Text

**Analysis of the data set from Schmidt et. al. including fast growth conditions** Due to the fast growth rate under LB and AA supplemented glycerol, compared with the other conditions measured in the data set from [29] these conditions were not included in our primary analysis as was noted in the Methods section. Including these conditions result in a much smaller set of proteins with a strong positive correlation with growth, as many of the proteins in that group in the slower conditions get down-regulated when AA are added to the media, significantly reducing their Pearson correlation with growth rate. For example, the Pearson correlation with growth rate of gapA, involved in glycolysis, drops from 0.73 to 0.35 when these conditions are included. Another such example is glyA, involved in Serine and Threonine metabolism, that has a correlation with growth rate of -0.12 when the faster conditions are included in the analysis vs. a correlation of 0.7 when they are excluded.

S5 Fig shows the implications of including the fast growth conditions in the analysis. As can be seen, many proteins are now less correlated with growth rate due to down regulation under the fast conditions. However, despite having fewer proteins being strongly positively correlated with growth (352 vs. 473) and despite the accumulated fraction of these proteins being lower under the slower growth conditions ($\approx 30\%$ vs. $\approx 40\%$), these proteins do occupy $> 50\%$ out of the proteome under fast growth in LB.
