# Supplementary material for: A Minimalistic Resource Allocation Model to Explain Ubiquitous Increase in Protein Expression with Growth Rate
Source: PLoS One. 2016 Apr 13;11(4):e0153344. doi: 10.1371/journal.pone.0153344 (PMC4830519; doi:10.1371/journal.pone.0153344)
Supplement: S7 Text — (PDF) [file pone.0153344.s012.pdf]

S7 Text

Discussion of reasons for differing ribosome concentration relation to growth rate Differences in ribosome concentration across growth rates as reported in different studies can result from a few factors:

1. Different growth rates and conditions monitored.
2. Inaccuracies and differences in the proteomic analysis procedures.
3. Usage of different strains.
4. In many studies the amount of ribosomes is deduced by measuring the RNA to protein ratio, assuming a relatively fixed portion of the RNA is rRNA. In our study, in contrast, ribosomal proteins are used as a proxy for estimating ribosomes concentration and, moreover, the RNA to Protein ratio is assumed to be constant. Therefore, and as it is known that ribosomes can operate even in the absence of some ribosomal proteins, such differences in manner of inference can account for some of the differences encountered.
