# Supplementary material for: A Minimalistic Resource Allocation Model to Explain Ubiquitous Increase in Protein Expression with Growth Rate
Source: PLoS One. 2016 Apr 13;11(4):e0153344. doi: 10.1371/journal.pone.0153344 (PMC4830519; doi:10.1371/journal.pone.0153344)
Supplement: S1 Table — (PDF) [file pone.0153344.s014.pdf]

S1 Table

Breakdown by function of strongly positively correlated with growth rate proteins in the data set from [29].

Function	Number of proteins	% of proteome	Correlated proteins	Correlated % of proteome
NotMapped	533	24.55	163	10.99
Carbohydrate Metabolism	113	20.9	21	4.53
Translation	110	13.81	81	12.67
Amino Acid Metabolism	92	9.48	41	7.62
Membrane Transport	70	6.49	5	0.31
Folding, Sorting and Degradation	97	6.07	37	3
Energy Metabolism	41	3.71	13	0.63
Nucleotide Metabolism	57	3.58	29	1.62
Transcription	48	2.93	12	0.27
Other enzymes	64	2.87	22	0.31
Lipid Metabolism	30	1.11	4	0.15
DNA maintenance	45	1.01	11	0.13
Metabolism of Cofactors and Vitamins	53	0.81	16	0.33
Metabolism of Other Amino Acids	19	0.8	7	0.48
Signal Transduction	28	0.64	2	$5.79 \cdot 10^{-3}$
Cell Motility	2	0.59	0	0
Cytoskeleton	5	0.3	2	0.13
Glycan Biosynthesis and Metabolism	13	0.22	3	$3.59 \cdot 10^{-2}$
Metabolism of Terpenoids and Polyketides	13	$8.75 \cdot 10^{-2}$	1	$1.36 \cdot 10^{-2}$
Not mapped	7	$2.79 \cdot 10^{-2}$	2	$3.15 \cdot 10^{-3}$
Xenobiotics Biodegradation and Metabolism	2	$1.96 \cdot 10^{-2}$	1	$1.79 \cdot 10^{-2}$
