# Supplementary material for: A Minimalistic Resource Allocation Model to Explain Ubiquitous Increase in Protein Expression with Growth Rate
Source: PLoS One. 2016 Apr 13;11(4):e0153344. doi: 10.1371/journal.pone.0153344 (PMC4830519; doi:10.1371/journal.pone.0153344)
Supplement: S2 Table — (PDF) [file pone.0153344.s015.pdf]

S2 Table

Breakdown by function of strongly positively correlated with growth rate proteins in the data set from [13].

Function	Number of proteins	% of proteome	Correlated proteins	Correlated % of proteome
NotMapped	387	23.86	67	8.81
Carbohydrate Metabolism	104	20.58	19	4.11
Translation	97	17.65	79	17.05
Membrane Transport	65	8.89	5	0.24
Amino Acid Metabolism	81	6.27	20	1.23
Folding, Sorting and Degradation	82	5.01	23	1.86
Energy Metabolism	41	4.28	19	2.79
Nucleotide Metabolism	47	3.62	30	2.9
Transcription	33	2.58	7	1.36
Other enzymes	46	2.11	5	$8.53 \cdot 10^{-2}$
Lipid Metabolism	18	1.29	5	0.59
DNA maintenance	33	1.14	5	0.17
Metabolism of Cofactors and Vitamins	39	0.72	8	0.25
Metabolism of Other Amino Acids	17	0.65	4	0.44
Cell Motility	5	0.43	1	$4.98 \cdot 10^{-2}$
Signal Transduction	23	0.31	5	$3.92 \cdot 10^{-2}$
Cytoskeleton	5	0.27	0	0
Glycan Biosynthesis and Metabolism	10	0.23	2	$7.49 \cdot 10^{-2}$
Metabolism of Terpenoids and Polyketides	7	$5.21 \cdot 10^{-2}$	1	$1.51 \cdot 10^{-2}$
Xenobiotics Biodegradation and Metabolism	2	$5.14 \cdot 10^{-2}$	0	0
