# Supplementary material for: A Minimalistic Resource Allocation Model to Explain Ubiquitous Increase in Protein Expression with Growth Rate
Source: PLoS One. 2016 Apr 13;11(4):e0153344. doi: 10.1371/journal.pone.0153344 (PMC4830519; doi:10.1371/journal.pone.0153344)
Supplement: S8 Text — (PDF) [file pone.0153344.s017.pdf]

S8 Text

Comparison of the passive resource allocation model to other recent models In [20–22] and [25], a sectioning model of the proteome is presented. According to these models, the proteome is roughly divided to sectors with the main sectors being a Q sector that is unaffected by growth rate, an R sector composed of ribosomal proteins and translation related proteins, and a P sector composed of metabolic proteins including catabolic and anabolic enzymes (the P sector can be further sub-divided according to the specific metabolic tasks groups of proteins perform). In this model, a specific protein may belong to more than one sector, where, for example, if protein P_i has a minimal value $P_i^{\min}$, then that fraction can be accounted for in sector Q, and only the changing fraction will be accounted for in sector R or sector P, according to P_i's function. The main point of the model is that different triggers may increase or decrease the expression of entire sectors, thus serving as global, or master, regulators. The model thus allows one to get an overview of the entire proteome as a result of the state of a few master regulators.

The model presented here takes a different approach. It does not classify proteins according to major classes, though it does not assume such classification does not exist. It mainly focuses on the consequences of actively regulating some proteins (be it a small number of highly expressed proteins, or a large number of proteins) on the proteins that are not being actively regulated. Our model therefore aggregates all non-actively regulated proteins between two specific conditions into one group, A, and all the regulated proteins between these two conditions into another group, B (where the inclusion of each protein in any of these groups depends on the specific pair of conditions considered). Our model then suggests that all of the proteins in group A, despite not being actively regulated, may still occupy different fractions out of the proteome between the two conditions, if the active regulation of the proteins in B changes the overall expression of B (noting that proteins in B may either be up regulated or down regulated, so their total sum may either increase, decrease, or remain the same between the two conditions observed).

To summarize, where the models from [20–22] and [25] focus on what classes of proteins exist, what are the master regulators, and how are they used to achieve optimal growth, the model presented here focuses on global patterns that passively emerge due to specific regulation on specific genes (be it regulation that is targeted at only a small number of genes, or a large group of genes), exposing the fact that positive correlation of protein fraction with growth rate can be an emerging property, not requiring active regulation of the positively correlated proteins.
